# Advances in Catheter Ablation of Ventricular Tachycardia: Technical Considerations and Contemporary Approaches

**DOI:** 10.31083/RCM52698

**Published:** 2026-08-17

**Authors:** Corrado De Marco, Alexios Hadjis, Wouter Saint-Phard, Jean-Marc Raymond

**Affiliations:** ^1^Division of Electrophysiology, Department of Medicine, Centre hospitalier de l’Université de Montréal, Université de Montréal, Montreal, QC H2X 0C1, Canada; ^2^Division of Electrophysiology, Department of Medicine, Hôpital du Sacré-Coeur de Montréal, Université de Montréal, Montreal, QC H4J 1C5, Canada

**Keywords:** ventricular tachycardia, catheter ablation, antiarrhythmic drugs, implantable cardioverter defibrillator, sudden cardiac death

## Abstract

Ventricular tachycardia (VT) is a life-threatening arrhythmia that most commonly arises from reentrant circuits within myocardial scar in patients with structural heart disease. Although implantable cardioverter-defibrillators (ICDs) reduce sudden cardiac death, these devices do not prevent VT and are associated with significant physical and psychological burdens, while antiarrhythmic drug therapy is often limited by incomplete efficacy and adverse effects. These limitations have driven the increasing use of catheter ablation to target arrhythmogenic substrates. This review examines the evolving role of catheter ablation in VT management, including the supporting evidence, patient selection, procedural timing, and contemporary mapping and ablation strategies, drawing on data from randomized clinical trials, observational studies, and recent technological advances. Multiple trials, including SMASH-VT, VTACH, VANISH, SURVIVE-VT, and VANISH-2, have shown that catheter ablation reduces VT recurrence and ICD therapies, with growing evidence supporting the earlier or even first-line use of this technique in selected patients. Substrate-based ablation has become the cornerstone of modern practice, enabling effective treatment during sinus rhythm; meanwhile, preprocedural imaging, such as cardiac magnetic resonance and computed tomography, enhances scar characterization and procedural planning. Advanced approaches, including epicardial ablation, bipolar and needle ablation, and alternative energy delivery techniques, help overcome the limitations of conventional endocardial radiofrequency lesions. Emerging modalities, such as pulsed field ablation, show promise but require further validation. Despite these advances, VT-free survival remains approximately 50–60%, reflecting the complexity of the underlying arrhythmogenic substrates. Overall, catheter ablation has become an integral component of contemporary VT management, offering meaningful reductions in arrhythmia burden and ICD therapies, with increasing use earlier in the disease course; meanwhile, ongoing innovations in mapping, imaging integration, and energy delivery are expected to improve outcomes further, pending continued investigation into optimal patient selection, procedural timing, and long-term efficacy.

## 1. Introduction

Myocardial scar represents the substrate in which ventricular tachycardia, a rapid and potentially life-threatening ventricular rhythm, develops. In patients with underlying structural heart disease, the predominant mechanism is macro–reentrant activation through bundles of surviving myocardium embedded within myocardial scar. These channels consist of strands of diseased tissue with slow conduction that traverse areas of myocardial fibrosis, forming the critical substrate required for reentry. Typically, reentrant ventricular tachycardia (VT) circuits are loops comprising an entrance, a central isthmus, and an exit site, which sustain repetitive activation. On the electrocardiogram (ECG), this manifests as a wide complex, often monomorphic and regular, tachycardia. Clinically, VT manifests as palpitations, presyncope or syncope, worsening heart failure, or, in severe cases, sudden cardiac arrest (SCA) and sudden cardiac death (SCD).

Prevention of SCD in patients with VT relies primarily on implantation of an implantable cardioverter-defibrillator (ICD) [1,2]. While highly effective at terminating malignant arrhythmias, ICDs do not prevent VT from occurring, but rather treat episodes once they occur using anti-tachycardia pacing (ATP) or high-voltage shocks. ICD therapies can be associated with significant physical and psychological burden. Shocks are often painful and distressing and may lead to anxiety and reduced quality of life [3,4]. Nearly a third of patients with an ICD will experience an appropriate shock in the three years following device implantation [5], and individuals with recurrent ventricular tachycardia requiring device therapy have increased mortality, more frequent hospitalizations for decompensated heart failure, and worse overall quality of life [6,7].

Pharmacological management with beta-blockers and antiarrhythmic drugs such as sotalol, mexiletine, dofetilide, and amiodarone has been used to prevent VT recurrences [8]. Although pharmacological therapies can reduce arrhythmia burden, they are frequently limited by incomplete efficacy and adverse effects. Amiodarone in particular is associated with substantial long-term toxicity, including pulmonary, hepatic, thyroid, dermatologic, and ocular complications [9,10,11], which can limit its tolerability and long-term use.

To directly target the underlying structural arrhythmogenic substrate, procedural approaches to VT treatment were developed. Early strategies involved surgical excision of subendocardial scar in patients with post-myocardial infarction VT refractory to medical therapy, which demonstrated efficacy in reducing inducible and spontaneous arrhythmias but was associated with significant operative risk [12]. Over time, less invasive catheter-based approaches have been developed. The percutaneous ablation of VT delivered high-energy direct current shocks through an intracardiac catheter and was reported in 1983 [13]. Subsequent advances greatly improved both the safety and effectiveness of these procedures. Techniques such as activation and entrainment mapping enhanced the understanding of reentrant circuits and allowed identification of critical components of the VT pathway where radiofrequency energy could be applied to interrupt conduction [14,15]. The development of three-dimensional electroanatomic mapping systems [16] further enabled detailed characterization of myocardial scar and facilitated substrate-based ablation strategies.

Clinical trials subsequently demonstrated the therapeutic benefit of catheter ablation. In the SMASH-VT study [17], patients with prior myocardial infarction undergoing secondary prevention ICD implantation who were randomized to prophylactic substrate-based VT ablation experienced significantly fewer ICD therapies compared with those receiving ICD implantation alone. Since then, continued improvements in mapping technologies, catheter design, and lesion delivery have enhanced procedural efficacy and safety [18,19]. Supported by an expanding body of clinical evidence [11,20], catheter ablation has therefore become an increasingly important component of the treatment strategy for ventricular arrhythmias. Nevertheless, the optimal timing and arrhythmia burden that should prompt initiation of suppressive therapies remain uncertain. This review examines current indications for VT ablation, considerations regarding procedural timing, and contemporary technical approaches used in modern VT ablation practice.

## 2. Literature Review

### 2.1 Evidence Supporting Catheter Ablation of Ventricular Tachycardia

Several randomized trials have evaluated catheter ablation for VT across different clinical contexts and time points in disease progression. Early studies such as SMASH-VT [17] and VTACH [20] demonstrated that adjunctive ablation in ischemic cardiomyopathy reduces recurrent ventricular arrhythmias and ICD therapies. SMASH-VT showed a significant reduction in ICD interventions (12% versus 33% in patients, *p* = 0.007) with prophylactic substrate-based ablation in patients with a history of VT at the time of ICD implantation, while VTACH demonstrated improved arrhythmia-free survival with ablation in patients with hemodynamically tolerated VT (47% at two years versus 29%, *p* = 0.045).

The SMS trial [21] did not show a significant reduction in recurrence of ventricular arrhythmia with prophylactic ablation, although ICD therapies were fewer in the ablation arm. In contrast, PAUSE-SCD [22], which included a substantial proportion of non-ischemic cardiomyopathy patients, demonstrated that first-line VT ablation reduced a composite outcome of VT recurrence, cardiovascular hospitalization, and death (HR 0.58 [95% CI, 0.35–0.96]; *p* = 0.04), driven primarily by decreased VT recurrence [22]. PAUSE-SCD, unlike most other trials analyzing the efficacy of VT ablation, included a large proportion of patients (65%) with non-ischemic cardiomyopathy.

VANISH was the first multicenter trial to compare catheter ablation directly with escalation of antiarrhythmic drug therapy in ischemic cardiomyopathy patients with recurrent VT despite treatment [10]. Ablation significantly reduced the composite of death, VT storm, or appropriate ICD shock (HR 0.72 [95% CI 0.53–0.98], *p* = 0.04), particularly among patients already receiving amiodarone, supporting ablation as an effective alternative to drug escalation.

Trials addressing timing have yielded mixed results. BERLIN-VT [23] found no difference in death or heart failure hospitalization between preventive and deferred ablation strategies, although early ablation reduced sustained VT and ICD therapies. PARTITA [24] suggested improved outcomes when ablation was performed soon after a first appropriate ICD shock, though event numbers were small.

More recent trials have further strengthened the role of ablation. SURVIVE-VT [25] demonstrated superiority of substrate-based ablation over antiarrhythmic therapy in reducing a composite of cardiovascular death, ICD shock, heart failure hospitalization, or severe treatment-related complications (HR 0.52, 95% CI 0.3–0.9, *p *= 0.021). VANISH-2 [11] further supported these findings in support of first-line ablation, showing that ablation was superior to initiation of antiarrhythmic drugs in reducing death and recurrent ventricular arrhythmias (HR 0.75 [95% CI 0.58–0.97], *p* = 0.028), with comparable safety profiles.

Collectively, these trials support catheter ablation as an effective strategy for reducing VT recurrence and ICD therapies, both after failure of medical therapy and increasingly as an early or even first-line intervention in ischemic cardiomyopathy.

### 2.2 Patient Selection and Timing of VT Ablation

Catheter ablation has emerged as an important therapeutic strategy for patients with ischemic cardiomyopathy and monomorphic VT. In appropriately selected patients, ablation may be considered as first-line suppressive therapy. The VANISH-2 trial [11] showed that first-line ablation significantly reduced the composite primary endpoint of death, VT storm, ICD shocks, and treated VT compared with antiarrhythmic drugs (sotalol or amiodarone). Notably, patients who were candidates for sotalol (generally better LV function and no VT storm) derived the greatest benefit from ablation (hazard ratio ~0.64 for the primary endpoint). In patients with more advanced structural heart disease or clustered arrhythmia events, outcomes following catheter ablation appear broadly comparable to those achieved with amiodarone therapy. However, the long-term safety profile of ablation relative to chronic amiodarone exposure remains an important consideration, particularly given the well-recognized systemic toxicities associated with prolonged amiodarone use.

Catheter ablation also plays a central role when antiarrhythmic drug therapy fails. For patients with ischemic cardiomyopathy having failed sotalol therapy, ablation represents a reasonable second-line suppressive strategy. When VT recurs despite treatment with amiodarone, catheter ablation is generally preferred given the limited therapeutic alternatives and the potential adverse effects associated with escalating antiarrhythmic drug therapy. Among all clinical presentations, patients with VT storm, defined as three or more sustained VT episodes within 24 hours, represent a subgroup with particularly compelling evidence supporting early ablation. In this high-risk population, catheter ablation has been associated with substantial reductions in recurrent arrhythmia, and, in some studies, improved survival compared with medical therapy alone [10].

The optimal timing of catheter ablation in the course of VT management remains an area of ongoing investigation, though recent data have provided additional clarity. The precise arrhythmia burden that should trigger initiation of suppressive therapy is not well established. Nonetheless, emerging data suggest that device therapies may serve as useful markers of escalating arrhythmic risk [24]. For example, episodes of appropriate ATP have been shown to predict subsequent ICD shocks, indicating progressive electrical instability [24]. Accordingly, contemporary practice increasingly considers catheter ablation after an appropriate ICD shock, a high burden of ATP, or the occurrence of VT storm.

In clinical practice, the timing of intervention should be individualized. Decisions often involve balancing the procedural risks of catheter ablation against the potential adverse effects of antiarrhythmic drug therapy and the morbidity associated with recurrent ICD shocks. Patient preferences and overall clinical context, including the degree of underlying ventricular dysfunction, comorbidities, and prior arrhythmia history, should therefore guide shared decision-making. Although current evidence supports a progressively earlier role for ablation in many patients with ischemic VT, further studies are needed to more precisely define the optimal timing of intervention, especially in patients experiencing VT in the context of non-ischemic cardiomyopathy.

### 2.3 Pre-Procedural Planning

Comprehensive pre-procedural planning is critical for successful and safe VT ablation. Initial evaluation should include detailed arrhythmia characterization with 12‑lead ECG of the clinical VT, if available, and ICD interrogation to identify the shock channel VT electrogram (EGM) morphology, especially in cases where no ECG is available.

Transthoracic echocardiography is routinely performed to quantify ventricular size and systolic function and to identify anomalies such as aneurysms, which often harbor the substrate in which VT circuits develop. Contrast echocardiography is particularly important to exclude left ventricular thrombus, which would preclude catheter access. Finally, thorough evaluation of both left and right ventricular function is essential, since severe biventricular dysfunction predicts higher procedural risk.

Advanced cardiac imaging often plays a central role in ablation planning. Cardiac magnetic resonance (CMR) with late gadolinium enhancement provides high-resolution scar characterization by delineating dense fibrosis, intermediate border zones, and surviving myocardial channels [26]. These imaging-defined channels often correlate with electrophysiologic slow-conduction zones identified at mapping [27]. Integration of CMR-derived scar maps into mapping systems allows a guided substrate approach. Multicenter registry data show that importing pre‑procedural late gadolinium enhancement (LGE)-CMR (and/or computed tomography (CT)) scar maps into the mapping system to guide scar dechanneling can significantly improve outcomes [28]. In one study, aggressive substrate ablation aiming to eliminate all arrhythmogenic scar (using image guidance) resulted in 72% of patients being non-inducible at procedure end, with only 25% VT recurrence over 18 months [29]. Cardiac CT can similarly highlight scar and ventricular wall thinning and is invaluable for defining epicardial anatomy (e.g., coronary arteries, pericardial adhesions) when an epicardial approach is considered.

Pre-procedural risk assessment is equally important, especially for minimizing procedure-related hemodynamic decompensation and cardiogenic shock. Many VT patients have advanced cardiomyopathy and limited functional reserve. Risk factors for acute hemodynamic decompensation during ablation include very low ejection fraction, severe heart failure (New York Heart Association (NYHA) class III/IV), chronic obstructive pulmonary disease (COPD), diabetes, and presentation with VT storm. Formal risk scores, such as the PAINESD [30] score, incorporate these variables to stratify patients’ peri-procedural risk. Recent work has shown that adding quantitative imaging markers to such risk scores improves risk prediction. For instance, a high total scar volume on pre-procedure CT was independently associated with approximately 5‑fold higher odds of peri-procedural circulatory collapse. Combining scar burden with PAINESD has led to the development of the PAINES2D [31] score, which has been shown to enhance prediction of instability beyond clinical factors alone, suggesting that imaging-derived scar metrics can inform preparation.

In patients deemed high-risk for hemodynamic decompensation, prophylactic hemodynamic support should be strongly considered. Mechanical circulatory assist devices, such as percutaneous ventricular assist devices, like the Impella, or veno-arterial extracorporeal membrane oxygenation, can maintain end-organ perfusion during prolonged VT mapping. These supports enable more extensive activation and entrainment mapping by allowing sustained VT even in compromised patients. Evidence suggests that the use of hemodynamic support may facilitate success in select high-risk patients, though these interventions carry their own risks (e.g., vascular complications, bleeding) and require careful team coordination [32,33,34]. In practice, most centers reserve prophylactic support for those with severely reduced ejection fraction (often <25%), biventricular failure, or VT storm at presentation.

### 2.4 Contemporary VT Ablation Techniques

#### 2.4.1 Identifying Targets for Ablation

Early catheter ablation strategies for VT relied on induction of the clinical arrhythmia followed by detailed mapping of the reentrant circuit during tachycardia. Activation and entrainment mapping were used to identify critical isthmus sites, and focal radiofrequency (RF) energy was delivered to terminate the arrhythmia. Although highly specific, this approach was limited by the fact that many VTs are poorly tolerated hemodynamically and cannot be sustained long enough to permit detailed mapping. As a result, contemporary VT ablation has evolved toward substrate-based strategies that allow identification of arrhythmogenic tissue during sinus rhythm or paced rhythm [35]. These approaches rely on electroanatomic mapping to delineate scar architecture and identify potential sites involved in reentrant circuits, followed by targeted ablation of abnormal substrate within the scar.

**Fig. 1.**
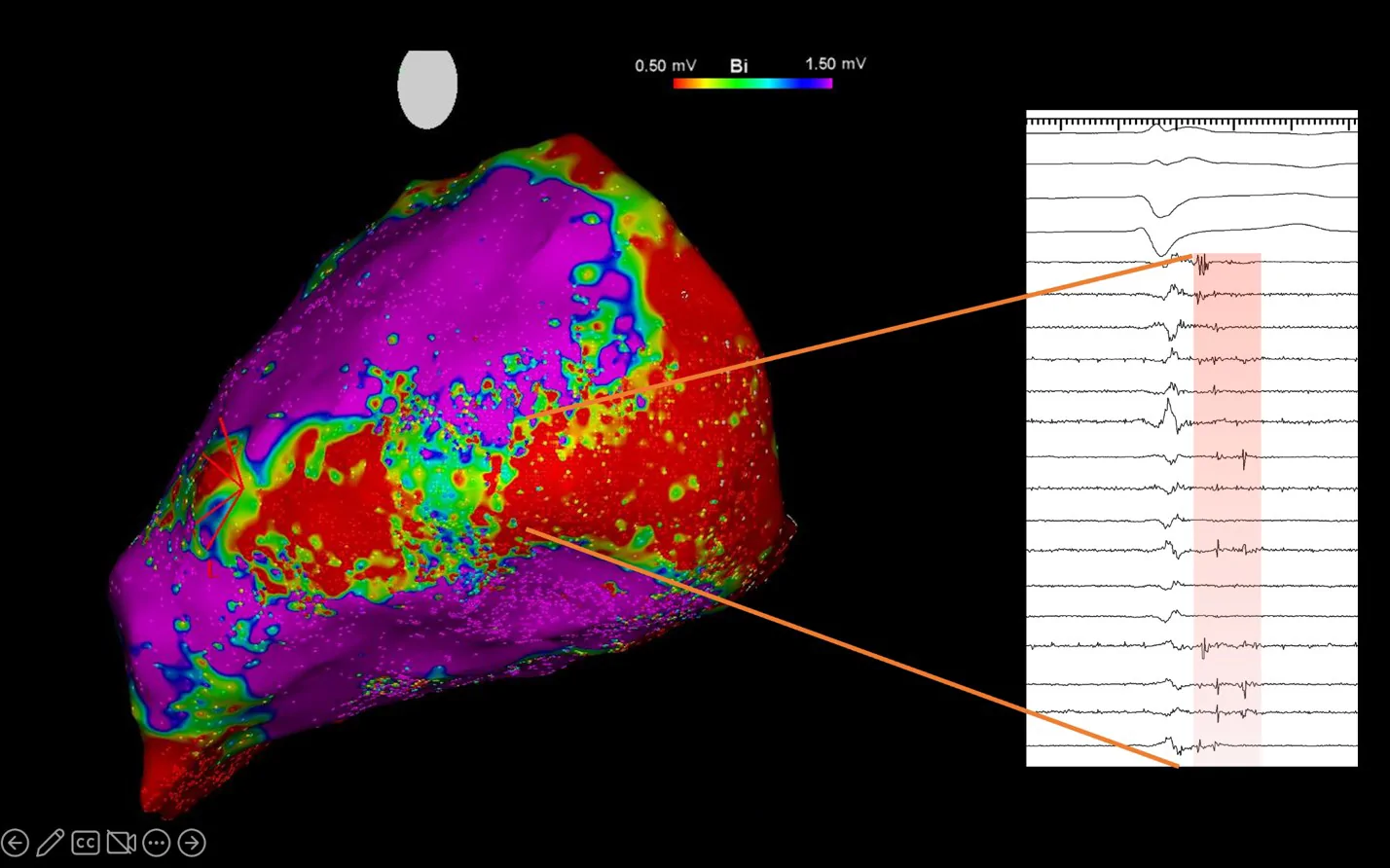
**Left ventricular bipolar voltage map created with the use of an electroanatomic mapping system**. Areas colored in red correspond to low voltage, designating diseased myocardium/scar, whereas voltages above 1.5 mV, colored here in purple, represent healthy myocardium. The scar area, defined by voltages <0.5 mV, demonstrates fractionated, late electrograms appearing after the offset of the QRS, indicative of potential arrhythmogenic substrate.

A randomized clinical trial demonstrated superior outcomes when substrate modification was performed in addition to targeting only clinically observed VT circuits [36]. Observational studies have further supported strategies aimed at ablating potentially arrhythmogenic substrate identified by mapping during sinus rhythm or with pacing. These approaches include ablation of regions with reduced bipolar voltage amplitude [37], elimination of late or highly abnormal ventricular electrograms [35] within scar (Fig. 1), and identification and modification of conducting channels embedded in dense fibrosis [38]. Functional interrogation of substrate has also been emphasized, with mapping strategies directed at areas of slow conduction [39], delayed late activation [40], and regions exhibiting decremental conduction properties [41]. More recently, substrate mapping techniques employing extra-stimulus pacing to unmask evoked delayed potentials have shown promise by identifying functionally relevant zones of slow conduction that serve as ablation targets [42,43].

Nonetheless, voltage mapping remains the cornerstone of substrate characterization. In electroanatomic maps, infarcted myocardium is typically identified by regions of reduced bipolar electrogram amplitude. Bipolar voltage <1.5 mV is commonly used to define abnormal myocardium consistent with fibrosis, whereas very low amplitudes (<0.3–0.5 mV) are often referred to as dense scar [44]. Electrogram amplitude reflects the amount of viable myocardium beneath the recording electrode and is influenced by several factors including electrode size, interelectrode spacing, conduction velocity, and catheter orientation relative to myocardial fibers. Importantly, the conventional 1.5-mV threshold may underestimate the true extent of myocardial fibrosis. Imaging studies comparing electroanatomic maps with cardiac magnetic resonance imaging have demonstrated that substantial scar may exist beneath a thin layer of surviving endocardial myocardium that generates bipolar voltages above this cutoff [45]. Unipolar recordings, which have a larger field of view, may therefore reveal deeper or epicardial scar not evident on bipolar mapping [46]. While extensive ablation of all low-voltage regions may increase the likelihood of eliminating potential culprit sites, such an approach may compromise specificity relative to techniques focused on defined or inducible circuits. Moreover, broad substrate homogenization increases procedural duration and cumulative RF exposure.

Greater specificity may be achieved through careful analysis of the surface electrocardiogram. VT morphology reflects the site of earliest ventricular activation, the “exit” site, and pace-mapping within scar can help delineate dense fibrosis and fixed lines of conduction block. Because the surface QRS morphology of VT largely reflects activation of myocardium outside the scar, pacing within the isthmus of a reentry circuit can reproduce the clinical VT morphology [47]. The presence of a stimulus-to-QRS delay during pace mapping indicates conduction through slow pathways within the scar before activation reaches the surrounding myocardium. Such delays may therefore identify sites within the reentrant circuit or its proximal isthmus, especially when pacing produces a QRS morphology identical to that of the clinical VT.

More selective ablation strategies target abnormal electrograms believed to represent surviving myocyte bundles within fibrotic tissue. Late potentials, manifesting as electrograms that occur after completion of the QRS complex during sinus or paced rhythm, are commonly identified within scar and may correspond to zones of slow conduction within reentry channels [35]. Elimination of late potentials during ablation has been associated with lower rates of VT recurrence [48], although these signals may also arise from bystander regions not essential to the reentrant circuit. Fractionated or multicomponent electrograms, particularly those with prolonged duration or multiple deflections, similarly suggest heterogeneous conduction through surviving myocardial bundles and are frequently targeted during substrate modification.

Recognition that abnormal conduction may be concealed during sinus rhythm has led to the development of functional substrate mapping techniques. Changes in activation sequence induced by pacing or programmed extra-stimuli can unmask delayed or fractionated electrograms that are not evident at baseline. These signals, variously termed evoked delayed potentials or decrement-evoked potentials, reflect conduction delay within surviving channels of myocardium embedded in scar [35]. Moreover, these late potentials can form wavefront discontinuity lines that have been shown to correlate closely with VT isthmus sites and may be more specific than conventional late potentials for identifying critical components of the reentrant circuit [49].

Isochronal late activation mapping (ILAM) has emerged as a complementary sinus rhythm–based strategy that does not require pacing or extra-stimuli. By constructing high-density activation maps during sinus rhythm and analyzing isochronal crowding, ILAM identifies regions of conduction slowing, termed deceleration zones, which represent areas of abrupt activation delay within the scar substrate [50]. These deceleration zones have been shown to co-localize with critical VT isthmus sites and may serve as practical targets for ablation [50], particularly in patients in whom VT is non-inducible or poorly tolerated.

**Fig. 2.**
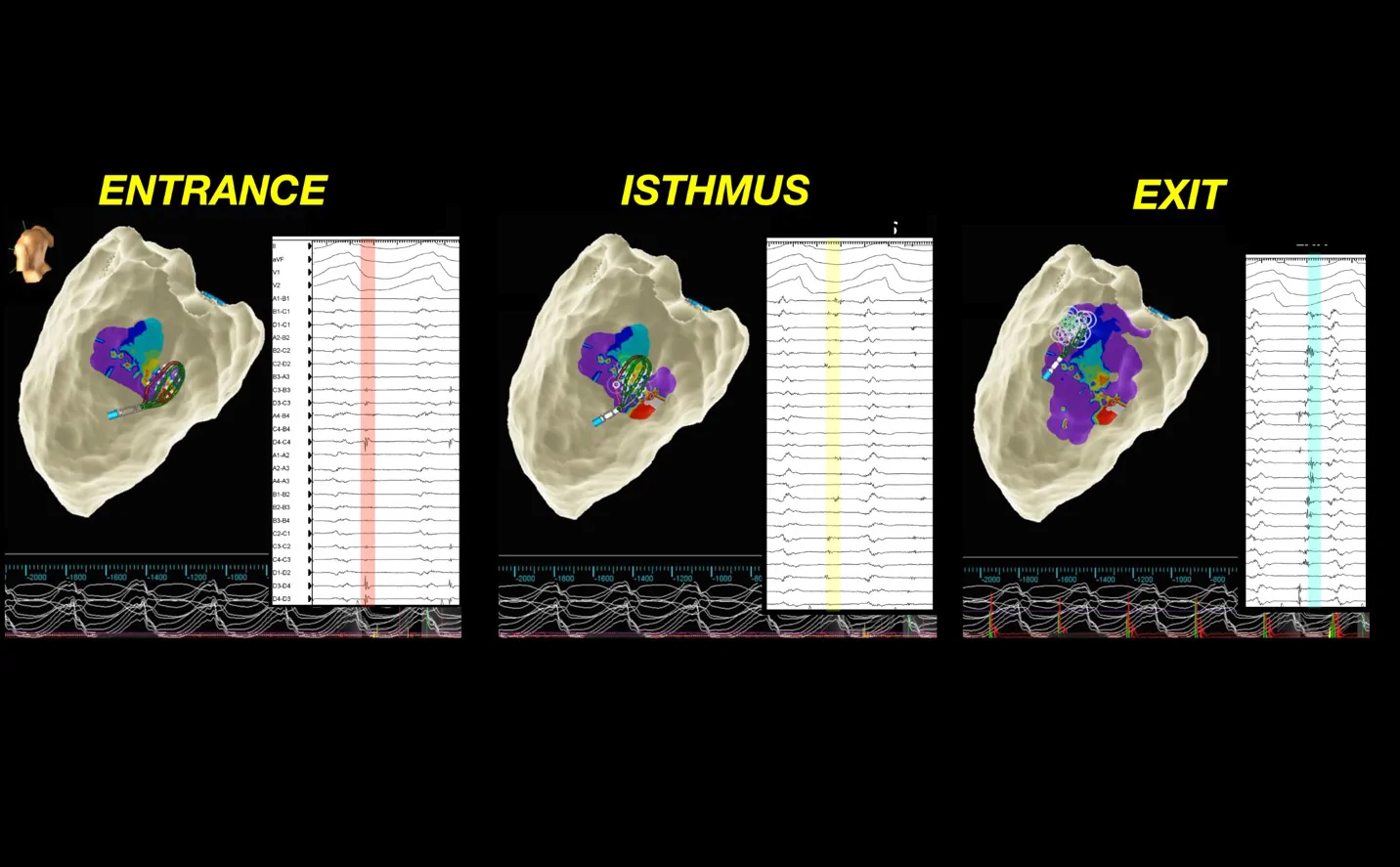
**A VT circuit broken down into its critical components from entrance (left) to isthmus (middle) to exit (right)**. Multipolar catheters can display EGMs from multiple points simultaneously and enable rapid identification of the different circuit components. VT, ventricular tachycardia; EGMs, electrograms.

Advances in high-density multielectrode mapping systems have further refined the identification of arrhythmogenic substrate, enabling rapid acquisition of extensive ventricular electroanatomic data. The ability to collect large datasets efficiently, even during sustained VT, has refined understanding of circuit architecture and facilitates meaningful activation mapping [51] (Fig. 2).

Despite these technical advances and improved procedural outcomes, long-term success rates may be approaching a plateau. Persistent challenges likely reflect circuits that remain undetected with current mapping strategies or arrhythmogenic substrates located beyond the depth or reach of standard endocardial lesion formation.

#### 2.4.2 Ablating VT Substrate

**Fig. 3.**
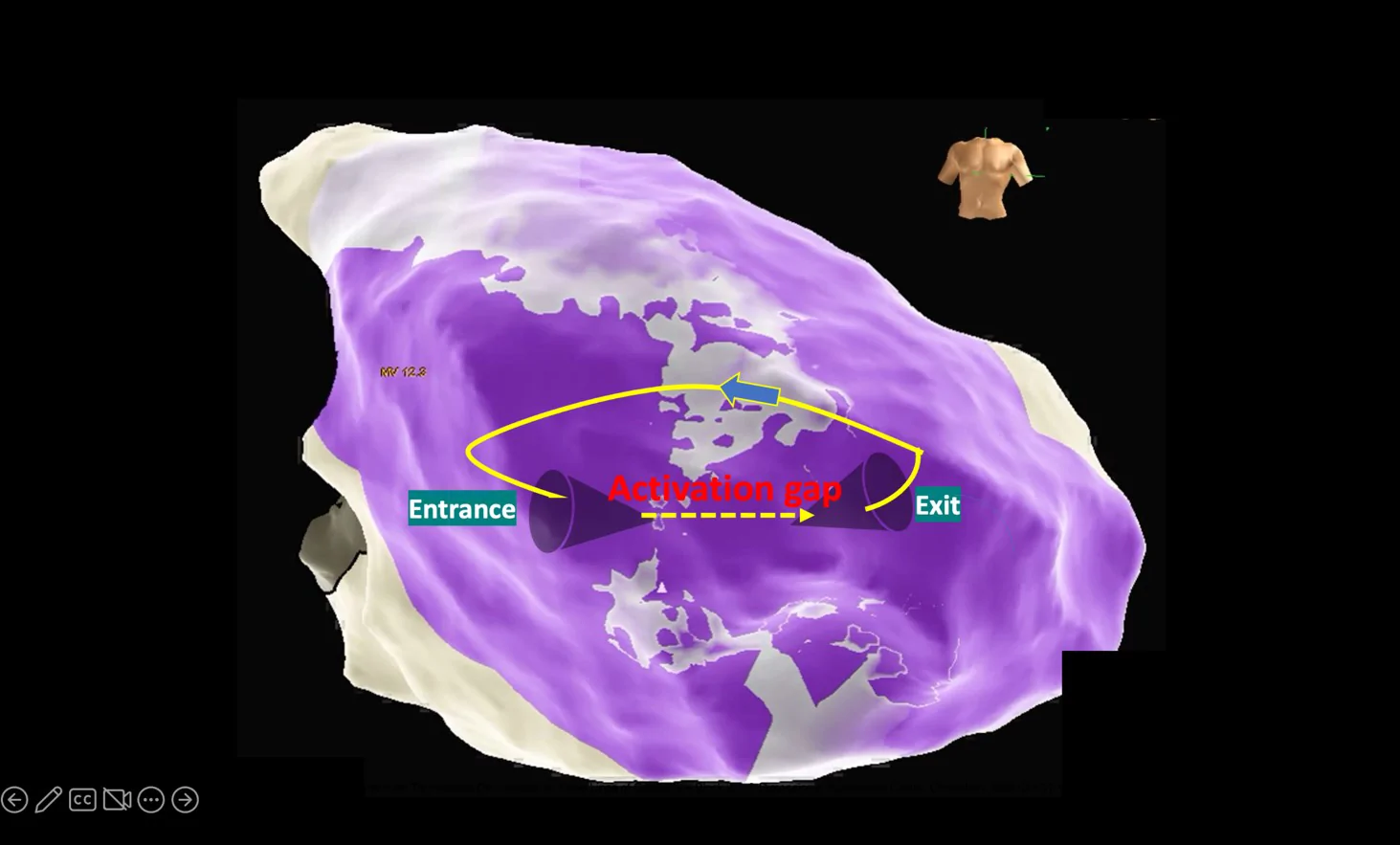
**VT propagation map demonstrating an activation gap at the level of the isthmus on the endocardial surface**. This pattern demonstrates the multiplanar nature of a significant proportion of VT circuits.

At present, irrigated RF catheter ablation with the use of an electroanatomic mapping system represents the standard approach for the treatment of VT. With contemporary techniques, VT-free survival following ablation has improved and is now reported in the range of 50–60% [52,53]. Activation mapping has played an important role in advancing the understanding of scar-related VT circuits, particularly highlighting their complex three-dimensional architecture and the frequent involvement of deep intramyocardial and epicardial components [54,55]. This three-dimensionality likely contributes to the historically limited success of ablation strategies targeting only the endocardial surface (Fig. 3). Indeed, intramyocardial mapping studies have confirmed the presence of deep circuit components that may not be effectively eliminated with conventional endocardial ablation techniques [56].

Recognition of the transmural nature of many VT circuits has led to increasing use of epicardial ablation as an adjunctive strategy, particularly in patients with ischemic cardiomyopathy. In a study of 89 patients with 151 VTs, non-uniform transmural propagation was observed in 61% of mapped tachycardias, and epicardial circuit components were present in up to 80% of ischemic VTs [55]. Clinical outcomes from epicardial ablation have been encouraging. In a multicenter study of 85 patients undergoing epicardial ablation for ischemic VT [57], most commonly as part of a combined endocardial–epicardial strategy, freedom from VT was achieved in 66.2% after a mean follow-up of 17.3 ± 18.2 months, with a substantial proportion of patients undergoing combined mapping from the outset. Improved pre-procedural identification of patients likely to harbor epicardial substrates may further refine procedural planning and improve outcomes. Nevertheless, epicardial access carries additional procedural risk such as coronary injury, bleeding, or pericarditis, with complication rates estimated between 4% and 7% [58]. Emerging techniques such as CO_2_ insufflation [59] to facilitate safe pericardial access and sustained apnea with right ventriculography [60] may help mitigate these risks.

**Fig. 4.**
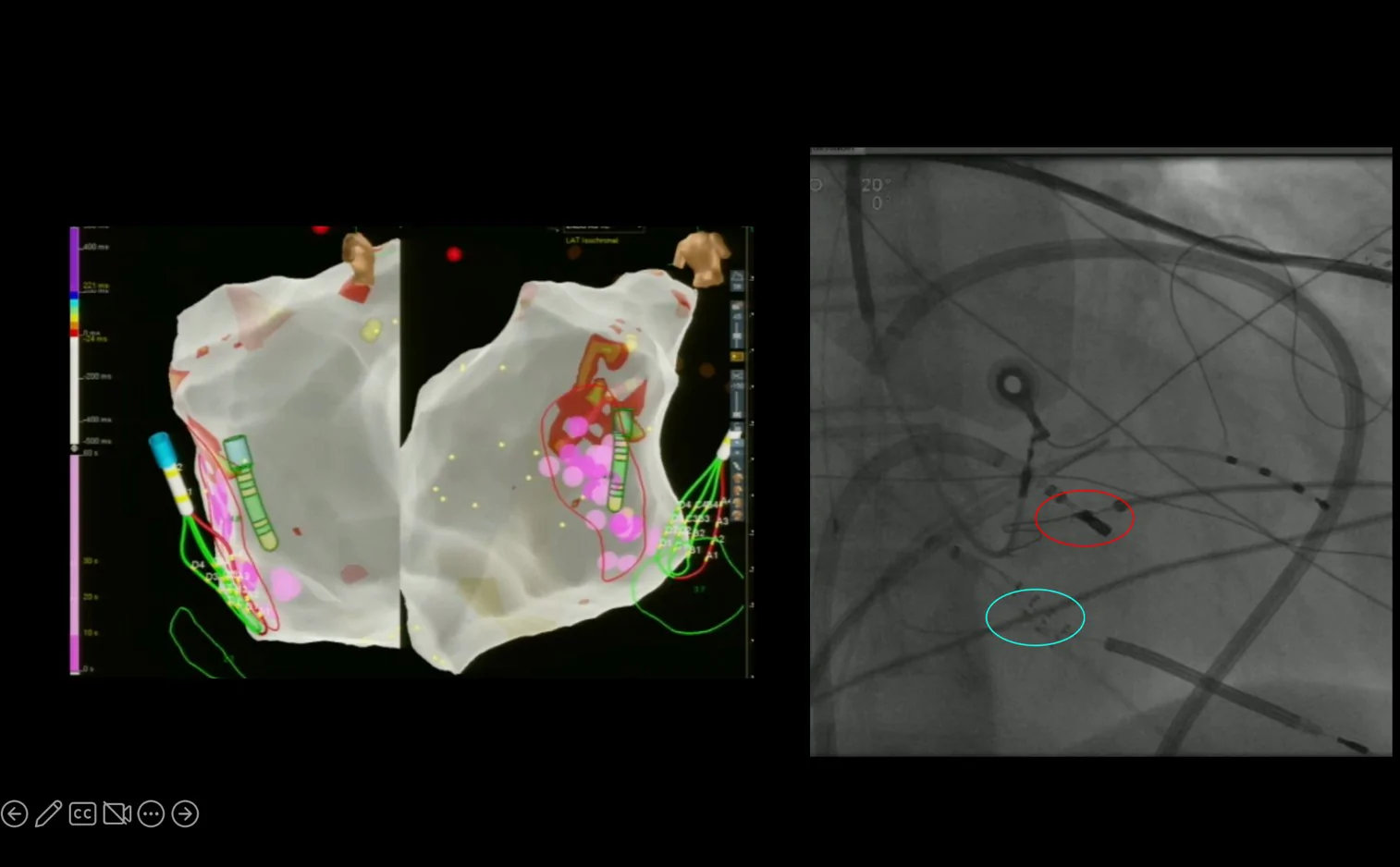
**Targeting mid-myocardial substrate with catheters placed on opposite sides of the myocardium**. The ablation catheter (red circle on fluoroscopy image) is positioned on the basal inferior LV endocardium while the mapping catheter (blue circle on fluoroscopy image) is positioned directly opposite on the epicardial surface, allowing for potential delivery of either bipolar or multipolar ablation. Both technologies have been evaluated as the development of systems designed to achieve deeper/transmural lesions progresses. LV, left ventricle.

The recognition that many VT circuits extend deep within the ventricular myocardium has also stimulated the development of technologies designed to achieve deeper or more transmural lesions. Lesion depths achieved by unipolar RF ablation are typically limited to approximately 6–8 mm [61], which may be insufficient for intramural substrates. Strategies aimed at increasing lesion depth include intramural needle ablation, which has demonstrated promising efficacy but is not yet commercially available. Bipolar RF ablation delivered between two catheters positioned on opposite sides of the myocardium has also been described and has shown feasibility and efficacy in multicenter series [62]. More recently, multipolar endo-epicardial ablation approaches have been reported to produce lesions with a high degree of transmurality [63] (Fig. 4).

Alternative methods for targeting deep or otherwise inaccessible substrates have also been explored. Transvascular ethanol infusion [64,65], delivered through arterial or venous branches supplying the arrhythmogenic region, has been used to eliminate refractory VT circuits when conventional ablation is ineffective, although the technique can be technically demanding. Additional strategies aimed at enhancing lesion depth include using half-normal saline as an irrigant during RF delivery [66] and ultra–low-temperature cryoablation [67]. Finally, stereotactic radioablation has emerged as a noninvasive option for patients with refractory VT and has shown promising early results, though its application must be balanced against the risks associated with high-dose radiation exposure and potential injury to surrounding structures [68].

#### 2.4.3 The Future of VT Ablation

Pulsed field ablation (PFA), a non-thermal ablation modality that induces myocardial cell death via electroporation, has emerged as a promising novel technology for VT ablation. Most significantly, PFA is known to have myocardial selectivity and has the potential to rapidly produce deeper, more homogeneous lesions in a lesser ablation time and with less collateral injury.

In the AVAAR study [69], investigators evaluated the safety and feasibility of ventricular arrhythmia ablation using a lattice-tip catheter capable of delivering both PFA and radiofrequency energy. The prospective multicenter registry included 126 patients with a high burden of disease, with most having structural heart disease and nearly two-thirds having undergone at least one prior VT ablation procedure. Procedural success was high, with 72% of patients being free from ventricular arrhythmia recurrence at 5.6 ± 3.7 months of follow-up. The study builds on earlier small reports which demonstrated early feasibility of PFA for ventricular arrhythmias but were limited by very small sample sizes.

The single-center, prospective CLEAR-VT [70] registry included 59 patients (mean LVEF ~36%), the majority of whom had scar-related VT. Acute procedural success, defined by non-inducibility of VT, was achieved in 78% of patients [70]. Over a median follow-up of approximately 100 days, VT recurred in 18% of patients, with an estimated VT-free survival of ~70%.

Despite these promising early findings, the experience of PFA in VT ablation remains limited. Follow-up has been short (~6 months), precluding assessment of long-term lesion durability and arrhythmia recurrence. In addition, over 60% of cases in AVAAR were redo ablations, introducing heterogeneity and limiting generalizability to first-time procedures. Similarly, major adverse clinical events in CLEAR-VT, including death, transplantation, or left ventricular assist device (LVAD) implantation, occurred in 12%, reflecting the advanced disease substrate. Finally, inconsistent use of pulsed field and radiofrequency energy across both studies complicates attribution of outcomes to the PFA modality itself.

Overall, PFA demonstrates technical feasibility and promise for VT ablation, but experience remains limited. Larger prospective randomized trials are needed to determine whether PFA offers meaningful advantages over established ablation strategies.

## 3. Conclusion

Catheter ablation has become paramount to contemporary management of VT in patients with structural heart disease. Advances in electroanatomic mapping, substrate-based approaches to both mapping and ablation, and peri-procedural imaging have substantially improved the ability to identify and target arrhythmogenic scar. Modern VT ablation increasingly relies on comprehensive pre-procedural planning, including integration of advanced cardiac imaging to delineate scar architecture, conduction channels, and anatomical constraints. These tools facilitate procedural planning, guide substrate modification strategies, and may improve procedural efficiency and long-term arrhythmia control.

Equally important are careful patient selection and procedural risk assessment. VT ablation is often performed in patients with advanced cardiomyopathy and significant comorbidities, requiring individualized evaluation of hemodynamic risk, potential need for mechanical circulatory support, and the need to strike a balance in assessing procedural risk and the morbidity associated with recurrent ventricular arrhythmias or antiarrhythmic drug therapy. Increasing recognition of high-risk features has led to more proactive strategies, including earlier referral for ablation and the selective use of hemodynamic support during mapping of unstable arrhythmias.

Critically, technological innovation continues to shape the field of VT ablation. Emerging energy sources, such as pulsed field ablation, offer the theoretical potential for deeper, more homogeneous lesions with greater myocardial selectivity, though their role in VT ablation remains to be defined. Early clinical experience suggests feasibility, but robust data evaluating long-term efficacy, lesion durability, and comparative outcomes with established radiofrequency ablation are still needed.

Despite these advances, VT ablation remains a technically demanding and evolving therapy. Continued innovation, coupled with rigorous clinical investigation, will be essential to refine contemporary approaches and further improve outcomes for patients with ventricular arrhythmias.
